# Supplementary material for: The historical range and drivers of decline of the Tapanuli orangutan
Source: PLoS One. 2021 Jan 4;16(1):e0238087. doi: 10.1371/journal.pone.0238087 (PMC7781382; doi:10.1371/journal.pone.0238087)
Supplement: Attachment — Submitted filename: Responses to editorial and reviewer comments.docx [file pone.0238087.s001.docx]

Dear Editor

Thank you and the reviewers for your comments and suggested edits.

We have taken feedback from all reviewers, as well as others who commented on the preprint version of the text on board. We significantly rewrote parts of the text, added new historic data and used this information to map the historic range of the species. For this we added one new figure to the text. This allows us to explicitly use the historic population declines and its drivers to inform today’s conservation management of the Tapanuli orangutan.

We also added the new IUCN Green Status framework and its principles for mapping species historic ranges and setting targets for restoring full ecological functionality of species. We believe this provides a more formal framework for understanding what species recovery would require. We also use insights from this to make the point that the Tapanuli orangutan is not a species adapted to high elevation conditions, but rather an ecologically flexible species now only surviving at higher elevations because it has been driven to extinction elsewhere.

We hope that you agree that this is much improved version of the text in which we address all the comments from the three reviewers as well as others who commented on the preprint manuscript.

Yours sincerely

Erik Meijaard

1. Please ensure that your manuscript meets PLOS ONE's style requirements, including those for file naming. The PLOS ONE style templates can be found at

https://journals.plos.org/plosone/s/file?id=wjVg/PLOSOne_formatting_sample_main_body.pdf and

https://journals.plos.org/plosone/s/file?id=ba62/PLOSOne_formatting_sample_title_authors_affiliations.pdf

**Response**: We consulted these two documents and edited the manuscript accordingly to bring it in line with the PLOS ONE formatting requirements.

2. We note that your author list was updated. Please complete our Authorship Change Form by following this link: http://journals.plos.org/plosone/s/file?id=13d0/plos-one-change-to-authorship-form.docx. You may return the form as an attachment by emailing plosone@plos.org or by uploading it as a submission file labeled with the file type ‘Other’. Please note that if your manuscript is accepted, we will not be able to complete the publication process without the completed form.

**Response**: We are already submitted this form in August 2020 and the authorship change was approved by the publishing editor Jordan Dacayanan (Case Number: 06769233)

3. Thank you for stating the following in the Competing Interests section:

"Several of the authors are engaged in programs focused on the conservation of the Tapanuli orangutan. Erik Meijaard and Serge Wich have actively advocated against a hydropower project that is being developed in the current range of the Tapanuli orangutan but have not received funding for this. These activities could be perceived to bias our research."

Please confirm that this does not alter your adherence to all PLOS ONE policies on sharing data and materials, by including the following statement: "This does not alter our adherence to PLOS ONE policies on sharing data and materials.” (as detailed online in our guide for authors http://journals.plos.org/plosone/s/competing-interests). If there are restrictions on sharing of data and/or materials, please state these. Please note that we cannot proceed with consideration of your article until this information has been declared.

**Response**: Done

Please include your updated Competing Interests statement in your cover letter; we will change the online submission form on your behalf.

**Response**: We included the revised Competing Interest statement in our cover letter to the chief editor as requested.

4. Please amend either the abstract on the online submission form (via Edit Submission) or the abstract in the manuscript so that they are identical.

**Response**: Done.

5. We note that Figures 1 and 2 in your submission contain map images which may be copyrighted. All PLOS content is published under the Creative Commons Attribution License (CC BY 4.0), which means that the manuscript, images, and Supporting Information files will be freely available online, and any third party is permitted to access, download, copy, distribute, and use these materials in any way, even commercially, with proper attribution. For these reasons, we cannot publish previously copyrighted maps or satellite images created using proprietary data, such as Google software (Google Maps, Street View, and Earth). For more information, see our copyright guidelines: http://journals.plos.org/plosone/s/licenses-and-copyright. We require you to either (1) present written permission from the copyright holder to publish these figures specifically under the CC BY 4.0 license, or (2) remove the figures from your submission:

**Response**: The country boundaries used in the map are from ESRI (esri.com). These are open for use: <https://www.esri.com/en-us/legal/copyright-trademarks>.

We digitized a 1950 forest cover map for Indonesia. The original copyright holder was Kolff & Co in Batavia. The company was nationalized in 1962 by President Sukarno to become the state owned enterprise PN Gita Karya (this is described in regulation Peraturan Pemerintah Nomor 24 Tahun 1962, which we can share).

According to regulation PP 47/1991, Gita Karya was incorporated into PT. Pradnya Paramita, which in 2004 was incorporated into PT Balai Pustaka (PP 34, 2004This makes PT Balai Pustaka the current copyright holder of this map. We contacted PT Balai Pustaka and they granted us permission to use the map.

All maps in this paper were created using ArcGIS® software by Esri. ArcGIS® and ArcMap™ are the intellectual property of Esri and are used herein under license. Copyright © Esri. All rights reserved. For more information about Esri® software, please visit www.esri.com.

**Review Comments to the Author**

Reviewer #1: This manuscript uses historical data to better understand the current distribution of the newest and most endangered great ape, Pongo tapnuliensis. This is a species of high interest and interesting results are presented using a new approach. I suggest publication after making the following revisions.

Did the authors look at museum records, specifically the catalog cards that show where specimens came from? These provide solid documentation of individual animals and where they came from. Many such records are available on-line or at least by contacting the museum. I think this would provide useful source of data that should be accessed for this paper that won't require too much additional work.

**Response**: Thank you for your positive feedback and suggestion to look at museum records. The lead author has worked on the taxonomy and morphology of orangutans since 2000 when he did his PhD with primate taxonomy specialist Professor Colin Groves. The lead author was also part of the team who described *Pongo tapanuliensis* in 2017. For this he worked closely (and supervised the PhD of) Dr Anton Nurcahyo who conducted a PhD on orangutan morphology. Between these three sources we have covered every relevant zoological collection in the world, either by visiting the collection and checking the catalogue or by contacting curators of the collections. As far as we are aware there are no other specimens of Pongo tapanuliensis, apart from those indicated in the manuscript (in the Leiden and Florence collections).

I also wonder how the authors distinguished between records of orangutans vs. gibbons/siamangs? "Orang pendek" (short person in Indonesian/Malay), who the authors reference in lines 164-169, is commonly thought to be a gibbon walking on the ground. I think the presence of gibbons/siamangs should at least be referenced as a confounder in this study.

**Response:** Potential confusion with orang pendek or other primates is indeed a concern. This concern was also raised by two ethno-zoologists who commented on the preprint version of our manuscript. On the basis of that input, we wrote a new section in the Discussion titled “The biases and constraints of a historical perspective.” We invite the reviewer to check this new text and see whether this sufficiently addresses the point of potential confusion between orangutans and other species. To make this point even clearer we added a column to Table 1 about the information type (i.e., specimen seen by the author, animal seen by the author, or anecdotal information), so the reader can better judge the reliability of each record.

It was a little unclear to me why some records were rejected and others weren't. For example, it would be hard to mistake a pig-tailed macaque from an orangutan (line 265). So, why were the records not accepted? The results thus seem a bit cherry-picked. It would be helpful to know that there was a systematic process to reject or accept the particular records.

**Response:** We indeed rejected Delmont’s claims, because the behaviour he described (catching groups of orangutans with cages) is very much unlike orangutan behaviour, and we think he was either bragging, or some information was lost in translation. Nevertheless, we agree that there is some subjectivity about judging the reliability and accuracy of individual records. As much as possible we have added text to justify why we accept some and reject other records.

One thing I thought was missing was an answer to the important question posed at the beginning of whether Pongo tapanuliensis is really a highland specialist. This wasn't really answered by the article.

**Response:** We agree that the original manuscript did not address this. In the revised text we have added additional historic records and mapped these in relation to the 750 m contour (the average elevation at which currently the species occurs). It shows that the species occurred across a wide range of different vegetation types, primarily in lowland, but also in upland areas. We now make the point in the discussion that the species does not appear to be an ecological specialist of higher elevations, and that this has consequences for restoring the full ecological functionality of the species.

It is thought that all species south of Lake Toba would historically have been Pongo tapanuliensis? This should be explained more.

**Response:** We have added text to the Discussion explaining that we cannot know for sure whether the orangutans south of Lake Toba were tapanuliensis or abelii. Genetic study of the two possible specimens from outside the current range (one in Leiden and one in Florence) could reveal this, but remains pending. Another interesting source of information would be the fossil orangutans in the Padang area, which through protein analysis could indicate closer affinity to either tapanuliensis or abelii. We do argue in the Discussion though that, given the general distribution patterns, it is more likely that orangutan south of Lake Toba are tapanuliensis rather than abelii.

The first figure should be labeled as Current Orangutan Distribution.

**Response:** Well noted, and we changed this.

Line 179: Figure 2 legend should be "orangutans 'were' historically..."

**Response:** Noted and changed.

Line 187: No need to say 'as the crow flies'

**Response:** Noted and changed.

Line 266: Should be 'were' summarized

**Response:** Noted and changed.

Line 294: Explain why it was though that these nests were thought to be eagle nests.

**Response:** Good question. When lead author Meijaard visited the area with Dr van Schaik and Dr Rijksen, both Sumatran orangutan experts, several nests were seen that were judged by all to have been built by an orangutan. Follow up surveys by co-author Wich could not confirm these orangutan nests but he did see nests high up on a tree that he judged to be eagle nests.

Line 466: should be "a" clear, science-based plan

**Response:** Noted and changed.

Reviewer #2: The manuscript reports historical evidence regarding the original range/distribution of the Tapanuli orangutan and discusses implications for conservation and management of the extant populations of this species. The historical occurrence information is of interest, but is limited in scope and quality. Because of the nature of the information sources, the authors had to speculate to some degree regarding which species was observed and the exact location of the observations. This limits inference. The discussion of the causes of range contraction are somewhat speculative. One would like to see additional information on human population growth and/or increased access to the forest which would support the apparent conclusion that hunting has been a contributor (major?) to population declines. The management implications discussed do not really depend upon the historical information; one would come to these conclusions simply understanding the current and recent trends in habitat loss and population declines of many Sumatran species. This species is clearly highly endangered and effective action is needed immediately. I am unclear to what extent the historical information adds to this argument.

**Response:** Thank you for your comments. We have revised the original text considerably, adding further historical records, noting caveats about accuracy and reliability, and mapping the historic range. This new analysis indicates that the current range of the Tapanuli orangutan is between 2.5% and 5% of the range in the 1890s and 1940s respectively. This indicates that the species has rapidly disappeared from large parts of its former range, mostly before large scale deforestation for agricultural development took place. We discuss in a revised text that the most likely drivers of this range contraction would have been fragmentation in lowland areas and highland plateaus for traditional agriculture and unsustainable hunting pressure. We also revised the text on implications for species conservation by adding text on current levels of fragmentation and take-off levels, making a case that the remaining three sub-populations of the Tapanuli orangutan continue to be under pressure from fragmentation and unsustainable take-off levels. Thus, we use the historic trends and likely drivers to inform today’s conservation challenges and management needs for addressing them.

The manuscript is generally well written but there are a few typos and inconsistent word usage. Sometimes the authors use Lake Toba while in other instances it is Toba lake. I prefer the word "elevation" to "altitude" in the context of heights on the Earth's surface. The authors use "extend" instead of "extent" at one point; clearly just a typo.

**Response:** Thank you for noting these errors. We have corrected the typos and changed ‘altitude’ to ‘elevation’ throughout.

The maps could be more clear, particularly Figure 1. And do the numbered locations in Table 1 correspond to the locations in Figure 2?

**Response:** We have improved the quality of the figures (and added a third figure) and we clarify that indeed the numbers in Table 1 correspond to those in Figure 2.

Regarding the discussion of historical accounts, I recommend that the publication date be mentioned each time a new publication is discussed. That will give the reader immediate historical context and eliminate the need for the reader to turn to the literature cited to determine publication date.

**Response:** That’s a good point and we have inserted the years of each of historic record in the text. We also show the years on the new Figure 3 so that readers can see how the population contracted over time.

The Tapanuli orangutan is critically endangered, and concerted conservation action is needed now. However, I believe that the information and analysis in the manuscript are not of sufficient interest or rigor to warrant publication in PLOS ONE. The authors could attempt to revise the manuscript to conduct a more rigorous temporal analysis of habitat loss and human hunting pressure, or they might seek publication in a different journal.

**Response:** Thank you for your critical comments. In the revised text we have strengthened the logical link between the historic analysis and current conservation challenges by mapping the historic range of the species based on additional new records that we found and by quantifying the range contraction since 1890 and 1940. While we recognize that there is uncertainty in the historic data, by now specifically addressing the caveats in the use of such information, we provide the reader with a better ability to judge the relevance of historic data for today’s conservation. The alternative of not using historic information would be to conclude that the tapanuli orangutan was always restricted to its current range – a conclusion we believe is wrong, also based on the fossil orangutans in the Padang area. We hope that the revised text makes a clearer case for this.

Reviewer #3: This article investigated the potential historic range of the recently described and critically endangered Tapanuli orangutan, including the likely habitat types historic populations occupied. They asked if the current range of this species is indicative of its historic range, and if not, is it possible to assess the reasons for the decline in range area. This topic is of particular immediate importance because all orangutan species, though especially those found on the island of Sumatra, are experiencing rapid population declines due to anthropogenic pressures. The authors used a historical ecology approach that combined written historical accounts from the past 130 years with a modern approach to spatial description. While the methods are overall qualitative, they are still appropriate for the question, and the scope of the submission fits within the requirements of PLOS ONE. They found that Tapanuli orangutans did indeed occupy a much larger range and potentially overlapped with the Sumatran orangutan. Additionally, the authors found that a combination of habitat destruction/fragmentation and hunting pressure resulted in the reduction of Tapanuli home range over the past century and a half.

In general, the manuscript is easy to read, uses a combination of methods in a unique, meaningful way, and conveys the importance of the topic. This project excites me and I believe it is a crucial addition to the literature on Tapanuli orangutans. Still, the manuscript requires revisions to better connect the aims with the results/discussion and to clarify several main points of the discussion.

**Response:** Thank you for your constructive feedback. We have taken feedback from all reviewers, as well as others who commented on the preprint version of the text on board. We rewrote parts of the text, added new historic data that we found and used this information to map the historic range of the species. For this we added one new figure to the text. This allows us to explicitly use the historic population declines and its drivers to inform today’s conservation management of the Tapanuli orangutan.

Major:

• In the final paragraph of the Introduction (lines 105-108), the authors should rework their aims statement. In its current state the statement is a method and not a research aim. Throughout the introduction, the main points of the project are mentioned, so these points should be explicitly stated as individual research aims.

**Response:** We have revised the final paragraph of the Introduction explicitly stating that “…. Based on the information we develop historical distribution maps as reference points for estimating historical population declines and their drivers, and better understand the ecological conditions under which the species used to occur. With this information we seek to inform current conservation strategies and provide data to conservation practitioners for setting long-term recovery targets for the species to ensure full ecological functionality.”

• In line 111 of the Methods, clarify which databases were used.

**Response:** We added this information.

• In lines 133 and 136-137 of the Methods, the authors describe designating vegetation types on a digitized historic map. However, vegetation types to this point have not been mentioned in the manuscript. The authors then continue to use vegetation types going forward. Therefore, the authors need to be more explicit and clarify how this method is used to address the states aims of the project.

**Response:** This is a fair point. In the revised text we discuss why understanding the vegetation types in which the species used to occur is important for conservation today.

• In lines 90-94 and 107 of the Introduction, the authors ask the question if Tapanuli orangutans are adapted to highland habitats and state it as a main aim of the paper, yet this is only limitedly mentioned in the discussion. The authors need to explicitly address this aim in the Discussion.

**Response:** By mapping the historic range in the revised version of the text (Figure 3), we have been able to estimate the extent to which the species occurred in upland and lowland areas. We have now incorporated this information in the Results and discuss the implications for conservation in the Discussion.

• In lines 359-360 of the Discussion, the authors state that historic populations of Tapanuli orangutans became extinct because of “…a combination of habitat loss and fragmentation, and mortality rates that exceeded reproductive replacement rates.” However, the authors in line 402 state the “… Tapanuli orangutan was hunted to extinction…”, but then again switch back to the main cause of extinction being habitat loss and fragmentation in lines 408-409. I believe the authors are saying that habitat fragmentation came first, and then the orangutans were hunted to extinction within these fragments. If this is the relationship the authors were trying to convey, then it needs to be more explicitly stated. If this is not the relationship they were trying to convey, then the authors need to reassess what the true cause of extinction was.

**Response:** While we added information by mapping the historic distribution ranges of 1890 and 1940, making it easier to visualize the amount of forest fragmentation that had taken place at that time, and we also added information about the likelihood that at least some people within that range were hunting and eating orangutans, the data do not allow us to say whether extinction was primarily driven by fragmentation or unsustainable mortality rates. We added text to the Discussion to convey this uncertainty: “… we recognize that in the complex socio-ecological system from which orangutans disappeared many processes may have contributed, and simple, linear cause-effect reasoning may not apply.” We did however point out that the combination of fragmentation and killing of females is especially problematic for the species because of the strong philopatry of females which makes it unlikely that females from nearby population will disperse into depleted populations. This drives a more rapid species decline with limited opportunity for recovery.

Minor:

• Throughout, fix “Tapanuli Orangutan” to “Tapanuli orangutan”

**Response:** We decided to consistently use the species name *P. tapanuliensis* throughout.

• Line 18 in Abstract: “…other literature” is very broad. Please be more explicit.

**Response**: We changed this to “newspaper, journals, books and museum records”.

• Line 86-87 in Introduction: The authors state that Tapanuli orangutans are found mainly around higher elevations (~834m ± 219m), but Nater et al. 2017 (reference 12 from manuscript) described the range from 300m-1300m. Need to clarify.

**Response**: The altitudinal analyses were based on more recent studies by co-author Wich (also co-author on the Nater et al. paper).

• Lines 125-127 in Methods: Clarify that the first official forest cover map is the 1950 Indonesia forest cover map.

**Response**: Noted and corrected

• Table 1: In Vegetation column, clarify if “(logged)” is historic or current and approximately when. If possible, include altitudes on Tall Swamp Forest habitats. Additionally, Point 13 has no value in Vegetation column.

**Response**: We clarified that the vegetation data were from Laumounier’s vegetation map of Sumatra dated 1997. There are no earlier vegetation maps in digital format that we can use. We agree that the term “logged” is confusing in that context, and have removed these terms from the table. We addressed point 13 (now 23) and added elevations for all vegetation types.

• Line 355-357: It is not clear what the 48% forested in the 1930s is being compared to. Please clarify.

**Response:**  Done
